# Bleomycin-induced flagellate erythema: A case report and review of the literature

**DOI:** 10.3892/ol.2014.2179

**Published:** 2014-05-26

**Authors:** HUI-YOUNG LEE, KYU-HYOUNG LIM, YOUNGJOON RYU, SEO-YOUNG SONG

**Affiliations:** 1 Department of Internal Medicine, Kangwon National University Hospital, Kangwon National University School of Medicine, Chuncheon-si, Gangwon-do 200-947, Republic of Korea; 2 Department of Pathology, Kangwon National University Hospital, Kangwon National University School of Medicine, Chuncheon-si, Gangwon-do 200-947, Republic of Korea

**Keywords:** bleomycin, skin toxicity, flagellate erythema

## Abstract

Bleomycin has been used most commonly in the treatment of Hodgkin’s lymphoma, certain germ cell tumors (GCT) and for the sclerosis of recurrent pleural effusions. Bleomycin toxicity predominantly affects the skin and lungs. Skin toxicity includes Raynaud’s phenomenon, hyperkeratosis, nail-bed changes and palmoplantar desquamation. Flagellate erythema is an unusual rash occurring specifically during bleomycin use. In the present study, we report a case of bleomycin-induced flagellate erythema in a patient with GCT. A 42-year-old male was diagnosed with stage IIIB testicular cancer and treated with bleomycin, etoposide and cisplatin chemotherapy. After 10 days from the initiation of treatment, the patient subsequently developed a generalized pruritus and erythematous linear rash that was most prominent on the trunk, and upper and lower extremities. The patient was commenced on a short course of low-dose oral prednisolone, 20 mg daily, and antihistamine. Consequently, bleomycin was withheld from the patient’s treatment regimen. The present study describes the case, along with a review of the associated literature.

## Introduction

Bleomycin is a chemotherapeutic antibiotic. Its mode of action is to block DNA uptake of thymidine in the S-phase of the cell cycle. Since it was first developed in Japan in 1966 (1), it has been used most commonly in the treatment of Hodgkin’s lymphoma, certain germ cell tumors (GCT) and for the sclerosis of recurrent pleural effusions (2). Bleomycin is inactivated in the majority of tissues by an enzyme, bleomycin hydrolase, which cleaves the ammonia group from bleomycin. This enzyme is active in all tissues, with the exception of skin and lung tissue, which may account for these being the most common sites of toxicity.

Reported dermatologic adverse effects of bleomycin include Raynaud’s phenomenon, hyperkeratosis, nail-bed changes and palmoplantar desquamation. Flagellate erythema is an unusual rash that appears as ‘whip-like’ linear streaks and occurs specifically during bleomycin use. Bleomycin-associated flagellate erythema has been reported since 1970 (3–5); however, with the declining use of bleomycin, this adverse reaction has become much less common in practice. In the present study, we report a case of bleomycin-associated flagellate erythema with a review of the associated literature.

## Case report

**Figure 1:**
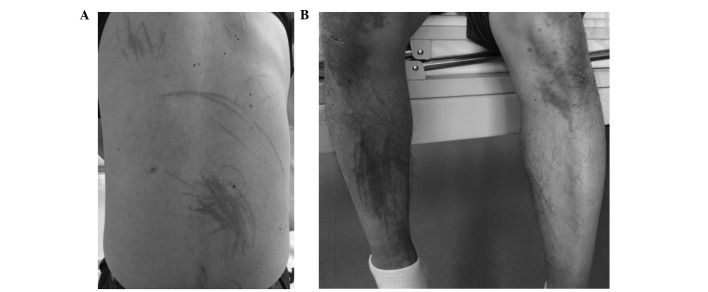
Multiple, well-dermarcated, erythematous patches in a linear configuration on (A) the back and (B) the lower legs.

In April 2013, a 42-year-old male was diagnosed with stage IIIB testicular cancer in accordance with the American Joint Committee on Cancer Staing Classification (6) at the Kangwon National University Hospital (Chuncheon, Korea). The histology revealed non-seminomatous germ cell tumor (NSGCT) comprising seminoma (60%), yolk sac tumor (25%), embryonal carcinoma (10%) and teratoma (5%). Following orchiectomy, serum α-fetoprotein (αFP) levels were 20 ng/ml and lactate dehydrogenase (LDH) levels were 298 U/l (normal, <190 U/l). Computed tomography (CT) scanning showed pre- and para-aortic lymphadenopathy ≤3.6 cm. There was no medical history of note and, specifically, no history of dermatological disorders or allergy. The pateint’s pulmonary function test results were normal. The patient was commenced on bleomycin, etoposide and cisplatin chemotherapy, intravenously from May 20, 2013 in the outpatient clinic. Bleomycin (30 units) was scheduled to be administered on days 2, 9 and 16, while etoposide (100 mg/m^2^) and cisplatin (20 mg/m^2^) were administered for 5 days from day 1. Treatment was intended to be repeated every three weeks. After 10 days from the start of treatment, the patient subsequently developed a generalized pruritus and erythematous linear rash that was most prominent on the trunk and upper and lower extremities. The patient visited the emergency room (ER) and was given antihistamine. The patient then revisited the clinic for bleomycin treatment on day 16, and the rash in which the patient appeared to have been whipped over multiple body areas was observed. Physical examination showed the appearance of an erythematous popular rash on the whole body, with evidence of dermatographia (Fig. 1). There were no scales or lichenification, and the patient’s vital signs were normal. Laboratory tests at ER showed a white blood cell count of 3,800/mm^3^ (normal range, 3,800–10,000/mm^3^) (segmented neutrophils, 70%; lymphocytes, 24%; and eosinophils, 3%), hemoglobin levels of 13.7 g/dl (normal range, 13.3–16.5 g/dl), a platelet count of 313,000/mm^3^ (normal range, 140,000–400,000/mm^3^), serum LDH levels of 208 U/l (normal range, <190 U/l) and C-reactive protein levels of 5.35 mg/dl (normal range, <0.75 mg/dl). Prothrombin time and activated prothrombin time were 11.5 (normal range, 12.1–14.5 sec) and 36.7 sec (normal range, 31.0–43.7 sec), respectively.

**Figure 2:**
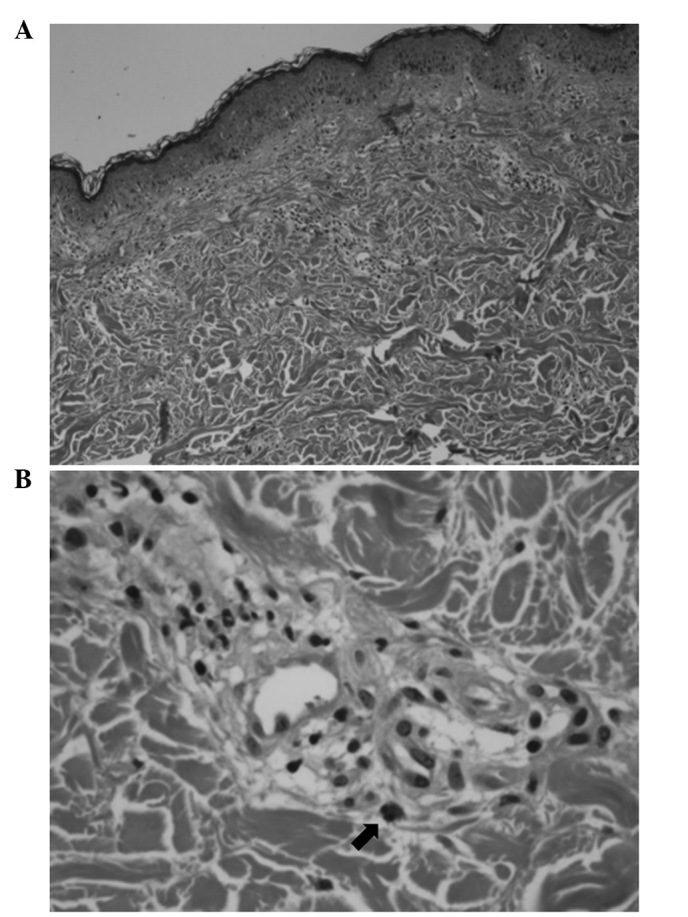
Histological findings of the skin (forearm). (A) Perivascular mononuclear cell inflammation in the dermis (stain, H&E; magnification, ×40). (B) Endothelial swelling and perivascular inflammation with eosinophils (arrow) (stain, H&E; magnification, ×400). H&E, hematoxylin and eosin.

A skin biopsy of a right forearm lesion was taken. Bleomycin treatment on day 16 was cancelled. Pathological examination of the skin biopsy showed endothelial swelling and perivascular mononuclear cell infiltration present in the superficial and deep dermis (Fig. 2). The findings were considered to be consistent with a systemic hypersensitivity reaction. Given the patient’s clinical history and the gross appearance of the lesions, the diagnosis was most compatible with a severe bleomycin-induced flagellate erythema reaction. The patient was commenced on a short course of oral prednisolone, 20 mg daily, and antihistamine. The itching sensation was improved, but mild hyperpigmentation remained.

Consequently, bleomycin was withheld from the treatment regimen. As the patient’s NSGCT was intermediate-risk, we recommended that the regimen should be changed to etoposide, ifosfamide and cisplatin. However, the patient refused to add ifosfamide following a discussion of the possible toxicities. Tumor markers, such as αFP and LDH levels, were normalized within 3 weeks of the first cycle of chemotherapy. Following a further three cycles of etoposide 100( mg/m^2^, days 1–5) and cisplatin (20 mg/m^2^, days 1–5), without bleomycin, every three weeks, no remnant mass was visible in the abdomen on positron emission tomography-CT.

## Discussion

Diverse cutaneous reactions to bleomycin therapy are common in the literature, and are reported as having an incidence of 8 to 20% in patients receiving cumulative doses >100 units. Bleomycin is associated with numerous dermatological toxicities, such as alopecia, skin ulceration (predominantly plantar-palmar), eczematous changes, erythematoid bulla, sclerodermoid lesions, nail-bed changes and Raynaud’s phenomenon (7). Flagellate erythema is a less common cutaneous toxicity of bleomycin, but is one with a strikingly characteristic presentation.

The development of flagellate erythema appears to be dose-independent, and flagellate erythema is considered to be a reaction specific to bleomycin and is independent of the route of administration or type of malignant disease being treated. The lowest reported dose with systemic dermatologic complications is 15 units given intravenously (8). Another report of low-dose bleomycin causing flagellate erythema involved the intrapleural administration of 30 units of bleomycin for the treatment of mesothelioma (9). Flagellate erythema may also occur at a dose of <15 units intracutaneously (10).

Several hypotheses regarding the cause of hyperpigmentation have been proposed. It has been proposed that the linear lesions are induced by rubbing or scratching the skin, which causes the drug to leak out of blood vessels. Alternatively, it has been suggested that accumulation of bleomycin in the skin causes a subsequent fixed drug eruption, due to the direct effects of bleomycin on the keratinocytes. Histopathologically, the lesions have shown a spectrum of morphological findings, including urticarial hypersensitivity reaction (4), localized increase in melanogenesis from hyperactive and enlarged melanocytes, inflammatory oncotaxis (8) and lymphocytic vasculitis (11).

The course of bleomycin-induced flagellate erythema is varied. The majority of patients initially develop generalized pruritus several hours to several weeks following the administration of bleomycin. Erythematous linear streaks eventually progress to the typical flagellate hyperpigmentation (8,9,12,13). Onset of the characteristic lesions can occur anywhere from 1 day to 9 weeks after bleomycin administration (14). There does not seem to be a characteristic distribution as cases have shown involvement of the face, trunk and extremities. Dermatographia is present to a limited extent and the role of scratching in producing the linear shape of the lesions has been debated (15). However, studies have shown the clear appearance of linear streaks in the absence of direct trauma (5). The majority of cases are reversible following cessation of bleomycin; however, persistence of hyperpigmented streaks for ≤1 year after treatment has been reported (8,9,12).

There is no specific treatment for flagellate erythema, which usually has a self-limited course of several weeks to months, as long as bleomycin is subsequently avoided, although permanent hyperpigmentation in affected areas is not unusual. Occasionally, topical corticosteroids with or without oral corticosteroids are required. Re-exposure to bleomycin may cause further extension or recurrence of this rash and should be stopped (16).

In summary, the present study describes a patient with flagellate erythema following bleomycin administration. Despite the declining use of bleomycin, clinicians should be aware of this peculiar cutaneous manifestation. Therefore, we have reported a characteristic feature of flagellate erythema with a review of the associated literature.
